# The Challenging Diagnosis of Pancreatic Masses: Not All Tumors Are Cancers

**DOI:** 10.1155/2015/832463

**Published:** 2015-04-05

**Authors:** Alessandro Morotti, Dario Gned, Leonardo Di Martino, Gaetano Cristaldi, Anna Alì, Paolo Nicoli, Andrea Veltri, Angelo Guerrasio

**Affiliations:** ^1^Department of Clinical and Biological Sciences, University of Turin and San Luigi Hospital, Orbassano 10043, Italy; ^2^Division of Internal Medicine, San Luigi Hospital, Orbassano 10043, Italy; ^3^Department of Diagnostic Imaging, San Luigi Gonzaga Hospital, University of Turin, Orbassano 10043, Italy

## Abstract

In the elderly patients, where biopsy-induced complications could outweigh the benefit, the identification of pancreatic masses is generally referred to as a synonymous of pancreatic cancer and patients are dismissed with no further options than palliative and supportive care. Notwithstanding, not all pancreatic tumors are cancers and therefore alternative diagnoses need to be investigated, especially when patients are unfit for invasive diagnostic procedures. Here, we report a case of an aged patient that was admitted to an internal medicine division for a previously diagnosed pancreatic cancer. The reassessment of the diagnosis has allowed identifying the pancreatic mass as a manifestation of focal pancreatitis in the context of an IgG4-related disease. Accordingly, patient was treated with steroids with rapid clinical improvement. This clinical case suggests that autoimmune diseases should always be considered in the differential diagnosis of pancreatic masses of the elderly.

## 1. Introduction

In the elderly, the identification of pancreatic masses is usually considered sufficient for the diagnosis of pancreatic cancer and therefore only palliative and supportive care is prescribed. According to several guidelines [1, 2], fine needle ago-biopsy is recommended only for patients with unresectable lesions to confirm the diagnosis and aid in decision-making regarding chemotherapy and radiation therapy. Therefore, in aged patients the risk-benefit assessment of this procedure and the lack of chemotherapy indications generally preclude any invasive biopsies. Notwithstanding, clinicians should consider that not all pancreatic tumors are cancers. In particular, clinicians should pose a diagnostic dilemma between untreatable pancreatic cancer and generally treatable focal chronic pancreatitis, with its miscellaneous presentations. Here, we report a case of a previously reported untreatable pancreatic tumor that, at the reassessment of the diagnosis, resulted in a focal pancreatitis in the context of an IgG4-related disease.

## 2. Case Presentation

**Figure 1:**
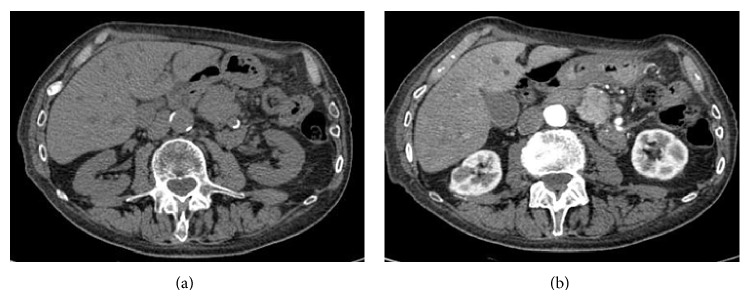
CT evaluation of pancreatic lesion. (a) Nonenhanced CT shows pancreatic body mass isodense to liver parenchyma; (b) pancreatic phase enhanced CT shows hypervascular pancreatic lesion.

**Figure 2:**
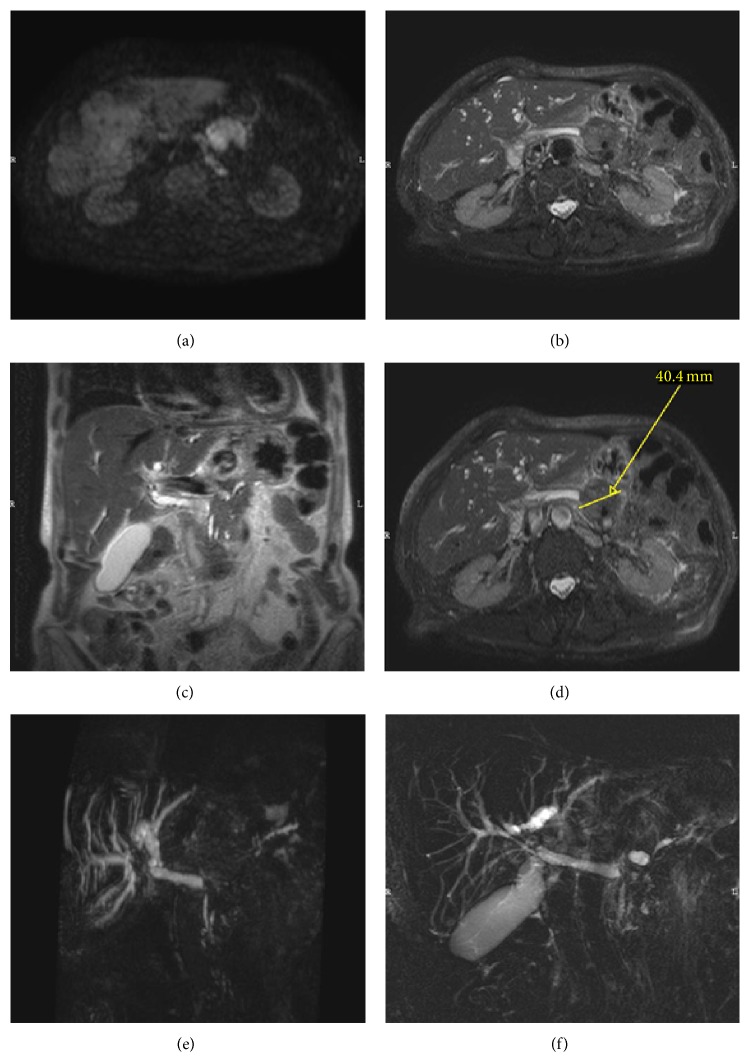
NMR evaluation of pancreatic lesion. (a) Diffusion weighted imaging (DWI) axial sequence shows a hyperintense pancreatic body mass with restricted apparent diffusion coefficient (ADC) value, confirmed in ADC map; (b) single shot axial T2-weighted fat sat image shows pancreatic body mass isointense to liver parenchyma; (c) single shot coronal T2-weighted image shows dilated extrahepatic biliary duct; (d) as shown in (b), with the indication of diameter of the lesion (4 centimeters); (e) 3D maximum intensity projection (MIP) coronal image showing dilated intra- and extrahepatic biliary ducts without evidences of middle segment of Wirsung duct; (f) T2-weighted radial big slab image of the same condition presented in (e).

In 2013, an 87-years-old male was admitted to an Italian peripheral hospital, suffering abdominal pain, weight loss, jaundice, and discomfort. A CT scan (not shown) of the abdomen was performed, revealing the presence of a pancreatic mass with diffuse infiltration of the rim of the organ and possible involvement of the stomach. This imaging approach was highly suspected for the diagnosis of pancreatic cancer. Due to the age of the patient and the compromised performance status, patient was considered unfit for pancreatic biopsy and was dismissed from the hospital. No specific therapy other than palliative therapy was offered. At home, patient's conditions were fluctuating over the following months and were characterized by emotional distress due to the presumptive diagnosis. One year later, patient presented with cholestatic jaundice (total bilirubin 7,2 mg/dL, direct bilirubin 5,4 mg/dL, alkaline phosphatase 588 I.U./L, and gamma-glutamyltransferase 342 I.U./L), bilateral swelling of the salivary glands, diffuse lymphadenopathies, and worsening of general clinical conditions. Therefore, he was admitted to the emergency department of our hospital and then transferred to our division of internal medicine, with the diagnosis of pancreatic cancer progression. However, the prolonged survival of this patient, in relation to the presumptive aggressive cancer diagnosis, and the overall performance status prompted us to reassess the diagnosis of pancreatic cancer. CT scan confirmed the presence of the pancreatic mass (Figures 1(a) and 1(b)). To better evaluate the nature of the pancreatic mass, a NMR was performed. As reported in Figure 2, this lesion was highly suspicious to be of inflammatory origin. Furthermore, the salivary gland swelling and the diffuse adenopathy of the patient were suggestive of an immunological disorder, with focal pancreatic involvement. Among the most likely diagnoses, we focused on the IgG4-related disease and Castleman disease [3]. Routine markers of autoimmunity (antinuclear antibodies, anti-dsDNA, and Reuma test) were negative, as well as pANCA and cANCA. However, high serum level of polyclonal immunoglobulin was observed (IgG 2922 mg/dL). Due to the age of the patient and the previous highly demanding history from the emotional perspective, we decided to avoid the patient's need for adenopathy or salivary gland biopsy and to limit the analysis to the dosage of immunoglobulin IgG4. The elevated dosage of IgG4 (IgG4 1070 mg/dL, (normal values 20–250 mg/dL)) was highly suggestive of IgG4-related disease. Patient received low dose steroids with sudden and dramatic improvement of clinical conditions, reversion of salivary gland swelling and adenopathies, and disappearance of abdominal signs/symptoms. After a follow-up of seven months, patient did not present any pathological symptoms and signs and the performance status was substantially improved.

## 3. Discussion

This clinical case clearly teaches us that not all pancreatic masses are synonymous of pancreatic cancer. This concept has relevant implications especially in elderly patients where the identification of a pancreatic mass is generally not further diagnosed with biopsies. If age or clinical status does not allow performing a biopsy of the pancreatic lesion, it should be highly recommended to evaluate all alternative diagnoses even if of rare occurrence. In particular, clinicians should always consider focal inflammatory lesions of the pancreas as alternative diagnoses [4, 5]. Here, we report the alternative challenging diagnosis of an IgG4-related disease. IgG4-related disease is an immune-mediated disease characterized pathologically by the infiltration of IgG4-bearing plasma cells with associated fibrosis into involved organs [6–11]. The histopathological hallmark of IgG4-related disease is a dense lymphoplasmocytic infiltrate characterized by at least 10 IgG4 positive cells per high-power field and organized in a storiform pattern [9]. Furthermore, obliterative phlebitis and eosinophil infiltrate are observed. These infiltrates commonly affect lacrimal glands, salivary glands, and pancreas, but other organs could display similar infiltration. In this patient, although the diagnosis was not supported by the lesion biopsy, IgG4-related disease was highly suspected on the basis of clinical and laboratory data (a pancreatic inflammatory disorder with involvement of salivary glands and adenopathies and high IgG4 serum level). Treatment generally involves low dose steroids with good response rates and prolonged improved quality of life [6, 7, 10, 11].
